# Assessing the impact of long term frozen storage of faecal samples on protein concentration and protease activity

**DOI:** 10.1016/j.mimet.2016.02.001

**Published:** 2016-04

**Authors:** Laura S. Morris, Julian R. Marchesi

**Affiliations:** a School of Biosciences, Cardiff University, Museum Avenue, Cardiff CF10 3AX, UK; b College of Medicine, Swansea University, Swansea SA2 8PP, UK; c Centre for Digestive and Gut Health, Imperial College London, London, UK

**Keywords:** Gut, Microbiota, Proteases, Proteins, Long-term storage, Enzyme activity

## Abstract

**Background:**

The proteome is the second axis of the microbiome:host interactome and proteases are a significant aspect in this interaction. They interact with a large variety of host proteins and structures and in many situations are implicated in pathogenesis. Furthermore faecal samples are commonly collected and stored frozen so they can be analysed at a later date. So we were interested to know whether long term storage affected the integrity of proteases and total protein and whether historical native faecal samples were still a viable option for answering research questions around the functional proteome.

**Methods:**

Faecal samples were collected from 3 healthy volunteers (3 biological replicates) and processed in order to be stored at both − 20 °C and − 80 °C and in a variety of storage buffers. Protein extraction, protein content and protease activity were assessed at the time of collection, after 24 h, 1 week, 1 month, 3 months 6 months and finally 1 year.

**Results:**

Beadbeating impacted the quantity of protein extracted, while sodium azide did not impact protease assays. Long term storage of extracted proteins showed that both total protein and protease activity were affected when they were stored as extracted protein. Intact faecal samples were shown to maintain both protein levels and protease activity regardless of time and temperature.

**Conclusions:**

Beadbeating increases the protein and protease activity when extracting from a faecal sample, however, the extracted protein is not stable and activity is lost, even with a suitable storage buffer. The most robust solution is to store the proteins in an intact frozen native faecal matrix and extract at the time of assay or analysis, this approach was shown to be suitable for samples in which, there are low levels of protease activity and which had been frozen for a year.

## Highlights

• Long term storage of protein in frozen faeces is viable.
• Protein concentration does not change if native samples are used.
• Protease activity is maintained in native frozen faeces.
• Both protein concentration and protease activity are reduced, in storage, once extracted from faeces.

## 1 Introduction

The human gut harbours microorganisms whose collective gene content outnumbers the host's own exome by at least 100 to 1 (Gill et al., 2006). While many studies have tried to understand interactions between bacteria and the host via a pathogen:host interactome model, we are currently exploring a commensal:host model and where disease appears, an amensal:host model. In the last two models the microbiota communicates with the host either by the metabonome or metaproteome. In order to make sense of the new form of dialogue many groups are implementing ‘omic’ based technologies, in particular, metagenomics, metabonomics and metaproteomics to elucidate the microbial functions responsible for the interactions, and the effectors which are derived from these functions. One such aspect of this molecular dialogue is the enzymes made by bacteria, which interact with host components to exert an influence on host functions, physiology and anatomy.

Faecal samples are often used as a surrogate for the gut microbiota (Eckburg et al., 2005) since they are considered an easy, safe and reputable proxy for describing both intra- and inter-personal differences in the gut microbiota composition and function (Peterson et al., 2008). In metagenomic studies DNA isolation is one of the most important steps and many parameters have been investigated which can affect both the quantity and quality of the nucleic acids extracted (Bertrand et al., 2005, Cowan et al., 2005, Ekkers et al., 2012, Purohit and Singh, 2009, Salonen et al., 2010). Long term storage of DNA extracted from faecal samples as well as other environments has also been extensively studied (McOrist et al., 2002, Salonen et al., 2010, Yuan et al., 2012). However, as the frequency of metaproteomic studies increases, so does the necessity to develop optimal protein extraction methods (Kolmeder et al., 2012, Lacerda and Reardon, 2009, Macfarlane et al., 1986, Tanca et al., 2014a, Tanca et al., 2014b). However, proteins can exist in many different biological forms, even if isolated from the same environment and so developing a generic extraction method for proteins is particularly difficult (Wilmes and Bond, 2006). Furthermore, protein samples for metaproteomics often do not need to maintain their activity, whereas clearly in activity-based studies or functional proteomics, this is of paramount importance. Therefore there is a clear need to understand the impact of storage regimes on the enzyme activity as well as the quantity of proteins in a faecal sample.

Protein degradation by microorganisms in the human gut has been linked with colon cancer (Hughes et al., 2000) and specific bacterial proteases have been shown to influence invasiveness of colon tumour cells (Flintoft, 2003). Microbial proteases represent approximately 5% of the genomes of infectious organisms (Shen and Chou, 2009) and approximately 2% of the genomes of commensals (Rawlings et al., 2014). Protease activity has been studied by direct methods; Macfarlane and colleagues have given us valuable insights into the functionality of the healthy gut (Macfarlane et al., 1986) and to some extent, the unhealthy gut as well (Steck et al., 2012). Most studies on proteases and how they may be impacting the host are carried out on pure cultures of specifically chosen microorganisms which may not actually be numerically dominant in the gut (Steck et al., 2011, Wu et al., 1998, Wu et al., 2007). While these studies are incredibly insightful, and demonstrate how significant microbial proteases and their interactions with host cells are, it is also useful to study enzymatic functions as a separate entity i.e. proteases as they are found in a particular environment at any one time point. Stool samples offer this non-invasive insight into proteolytic activity of the human gut microbiota as it may be occurring in situ.

Thus the main aim of this work was to assess the parameters that may introduce variability upon storage of faeces for protein and protease studies. In this study, we tested a variety of variables which we hypothesise might influence protein stability and thus protease activity, these included, buffer composition, time, temperature, extraction method and preservatives.

## 2 Materials and methods

### 2.1 Buffer composition and preparation

All buffers were made up in molecular grade water (18 MΩ) and where possible steam sterilized at 121 °C for 15 min, where heat labile ingredients were present; buffers were filter sterilised (0.2 μm). Two buffers were chosen for futher assessment, and the composition for each one is shown in Table 1. All reagents used were available from Sigma (Poole, UK) unless otherwise stated.

### 2.2 Collection of faecal samples

Assays to detect and quantify enzymatic activities were developed and optimized with faecal specimens from laboratory volunteers (n = 3). Institutional ethical approval was obtained prior to the study and was granted by Cardiff School of Biosciences, Cardiff University (Cardiff University's Research Ethics Committee). Participants gave written consent following retrieval of a participant information sheet detailing all aspects of the study and for what their samples would be used.

Faecal samples were collected from healthy male participants from Cardiff University. All samples were processed and stored following correct protocol as determined by the Human Tissue Act (HTA) 2004 and samples were all anonymised. Samples were collected using disposable trays; participants were advised to ensure that no urine was collected in the tray alongside the faecal sample. Trays were deposited anonymously in a fridge at 4 °C until DNA extraction (the same day). The remaining sample was thoroughly mixed and divided into 1 g samples in sterile universal tubes and immediately frozen at − 20 °C and − 80 °C.

### 2.3 Protein concentration measurements and protease assays

Protein concentrations were assessed using the bicinchoninic acid assay according to the manufacturer's instructions (PIERCE, Rockford, IL, USA). General protease activity was determined by measuring the release of acid-soluble substance from azocasein (Sigma-Aldrich) over a period of 3 h after precipitation. Azocasein (5 mg/ml) was prepared in 50 mM Tris–HCL, pH 8. In order to assess protease activity in total protein samples (100 μl at 1 mg/ml protein (vide infra)) were added to the azocasein solution (100 μl). The mixture was incubated at 37 °C and the reaction was terminated by the addition of 400 μl of 10% (w/v) trichloroacetic acid (TCA). Protein was precipitated by centrifugation at 12,000 ×* g* for 5 min and the resulting supernatant was transferred to a clean tube containing 700 μl 525 mM NaOH. The absorbance was measured using a spectrophotometer at 442 nm. Each reaction was carried out in triplicate. Negative controls were prepared by setting up a reaction and immediately terminating the reaction with TCA. The resulting precipitate was taken as a negative control. To minimise background interference, a further negative control was set up with just water. Proteinase K (Sigma Aldrich) was used as a positive control at a concentration of 2 μg/ml.

### 2.4 Design, conduct and analysis of storage media for evaluation of protein yield and protease activity over time

The experimental process is shown in supplemental Figs. S1 and S2. To expand on this, fresh material from one faecal sample (collected from 3 healthy volunteers in total) was collected and divided into 4 subsamples (1 g). One sample for each buffer and each sample would be used for total protein extraction (i.e. with bead beating) and for extracellular protein only analysis (no bead beating) each containing 1 g faecal material. Each sample was allocated to a buffer (1, or 2) and this buffer was added to the faecal sample to prepare a 10% w/v faecal slurry which was homogenised by mixing on a Vortex Genie 2™ until no clumps remained. To prepare the crude total protein extract the faecal slurry was divided into 2 ml RNAse and DNase free lysing matrix E tubes (MP Biomedicals) containing 1.4 mm ceramic spheres, 0.1 mm silica spheres and one 4 mm glass sphere. Samples were kept on ice throughout. The samples were subject to bead beating using a FastPrep-24 bead beater (MP Biomedicals) at a speed of 6.0 m/s for 30 s with a period of 5 min on ice between each beating. To determine the optimal number of bead beating steps this process was repeated up to 6 times. The bead beating step was repeated further 2 times for optimal recovery of intracellular protein. Samples were subject to centrifugation at 20,000 × g for 30 min at 4 °C and the supernatant from this step was filtered through a 100k Amicon Ultra centrifugal filter tubes (Millipore, Darmstadt, Germany) according to the manufacturer's instructions to allow proteases through. For the extracellular only samples, this centrifugation step was conducted immediately instead of the bead beating step. Supernatant after filtration was transferred to new sterile tubes and taken as the crude protein extract. Sodium azide (NaN_3_) was added aseptically to each sample to a final concentration of 0.05% w/v. Samples were divided into 1 ml aliquots and stored at − 20 °C. Neat samples, 10-fold and 100-fold dilution were used to estimate protein concentration using the bicinchoninic acid assay (BCA) method according to the manufacturer's instructions (PIERCE, Rockford, IL, USA) and samples were normalised to 1 mg/ml protein using the appropriate buffer as a diluent to conduct subsequent protease activity estimates. Azo-casein assay was performed as described above. The protein concentration measurements and protease activity estimates were performed on the aliquoted samples after 24 h, 1 week, 1 month, 3 months, 6 months and 1 year.

### 2.5 Design, conduct and analysis of the effects of storage of whole faecal samples at − 20 °C and − 80 °C

To assess whether a period of one year of storage of faecal samples provides impacts on the reproducibility on measurements of protein concentration and protease activity, faecal samples from 3 healthy volunteers were collected. Each sample was thoroughly mixed in a sterile environment and 13 lots of 1 g specimens were separated into sterile containers. 1 sample from each individual was processed immediately, and 6 samples from each individual were stored at − 20 °C and the remaining 6 were stored at − 80 °C. The fresh sample was subject to crude protein extraction, protein measurements and protease assay (vide supra). The frozen samples were analysed in exactly the same way after 6 storage time points; 24 h, 1 week, 1 month, 3 months, 6 months and 1 year. Percentage changes in protein concentration or protease activity were calculated by taking the data for the fresh faecal extracts as 100% and comparing all subsequent data to this value. This approach overcomes the variability in the protein concentration and protease activity inter-individually.

### 2.6 Statistical analysis

Differences between the buffers and differences between the storage of faecal samples at sub-zero temperatures in reproducibility of mean protein concentration were determined by correlation analysis using the Spearman method and the pairwise comparison using the students *t*-test using a Holm adjustment for familywise error after the samples had been demonstrated to follow a normal distribution. The same statistical analysis was implemented to compare the mean protease activity over time. To determine optimal bead beating steps mean enzyme activities were compared with pairwise comparisons using the Wilcoxon signed rank test with a Holm adjustment method. Similarly to determine the effect of NaN_3_ mean enzyme activity was compared using the Wilcoxon signed rank test. All statistical analysis was conducted in R software.

## 3 Results

### 3.1 Effect of number of bead beatings on protein concentration and protease activity

**Fig. 1:**
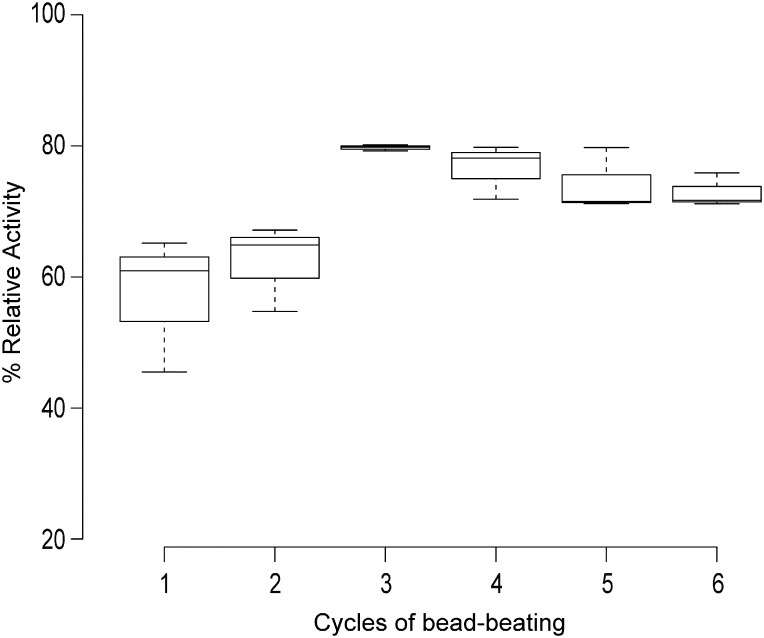
A boxplot of the mean of triplicate data + standard error of the means. Statistical significance was determined by non-parametric multiple comparison testing using a Holm adjustment method to control for familywise error rate. Significantly higher protease activity was observed after 3, 4, 5 and 6 rounds of bead beating (P = 0.0065, 0.0196, 0.0430 and 0.0639 respectively) when compared to 1 round of bead beating. Significantly higher activity was also observed after 3 rounds of bead beating compare to 2 rounds (P = 0.0377).

The effect of the number of bead beating steps on protease activity is shown in Fig. 1. Results represent the mean ± S.E.M of triplicate results from participants. Protein concentrations were significantly different after 3, 4, 5 and 6 bead beating steps (data not shown, P < 0.05) when compared to the concentration after one beating. Protease activity was also significantly higher after 3 beatings when compared to 2 beatings however no difference was observed after 4, 5 and 6 beatings compared to 2 beatings, suggesting that more than 3 beatings may be deleterious to protease activity.

### 3.2 Effect of sodium azide (NaN_3_) on protease activity

**Fig. 2:**
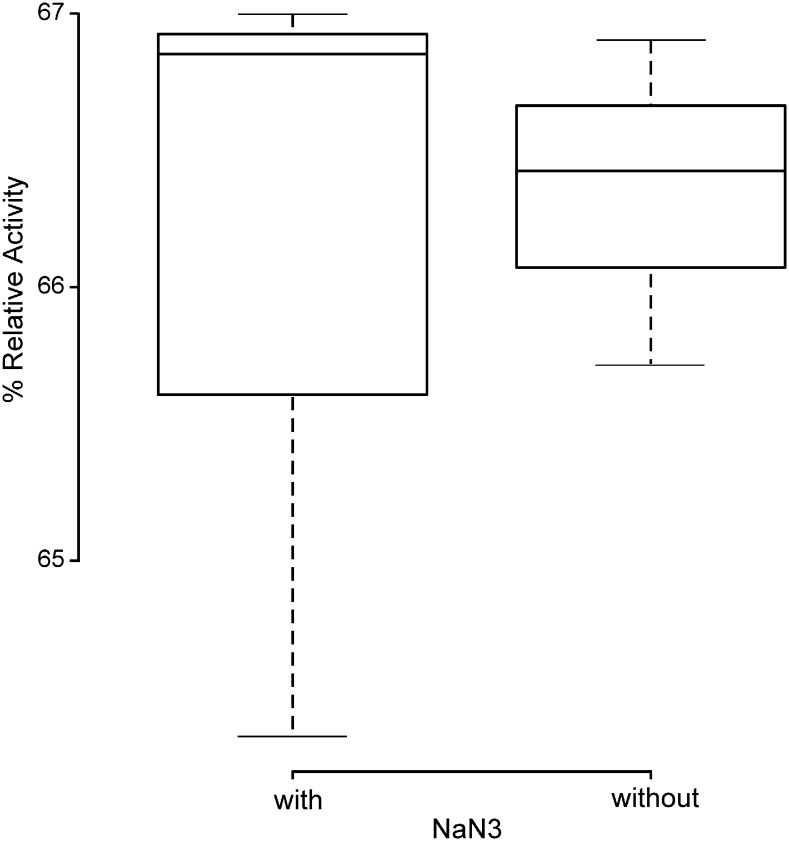
Effect of 0.05% w/v NaN_3_ on protease activity. Centre lines show the medians; box limits indicate the 25th and 75th percentiles; whiskers extend 1.5 times the interquartile range from the 25th and 75th percentiles. Statistical significance was determined by the student's *t*-test. (P > 0.05).

The effect of NaN_3_ on protein concentration after extraction and protease activity was determined by conducting an azo-casein hydrolysis assay from a normalised input of protein (1 mg/ml). To remove bias from differences in protease activity between individuals, % relative activity was calculated separately for each sample based on 100% activity being the hydrolysis of azocasein after doubling the incubation time (4 h). No significant difference was found between protease activity (Fig. 2) when NaN_3_ was used and when it was absent. Thus we concluded that the presence of this preservative does not affect these measurements, at the concentrations used and so can be used in buffers to aid in long term storage of extracted faecal proteases.

### 3.3 Stability of extracted proteins and protease activity over time

**Fig. 3:**
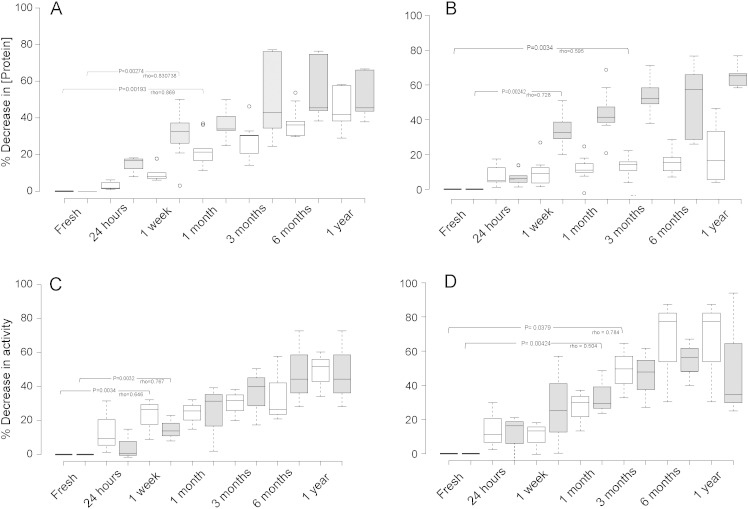
(A) The relationship between length of storage and the % decrease in protein concentrations of total protein extract after bead beating (total protein) in two different buffers; grey = buffer 1 and white = buffer 2. (B) Levels of extracellular protein after storage. (C) The relationship between length of storage and the % decrease in protease activity in total protein samples stored in the two different buffers and normalised to 1 mg/ml. (D) Extracellular protein protease activity. Centre lines show the medians; box limits indicate the 25th and 75th percentiles; whiskers extend 1.5 times the interquartile range from the 25th and 75th percentiles, outliers are represented by dots. n = 9 sample points.

To monitor the reproducibility of protein concentration estimates after extraction and storage over time, a crude protein extract was prepared in triplicate, from fresh faecal samples from 3 individuals. All samples exhibited a significant correlation between the decrease in protein concentration and storage time, regardless of buffer type (Fig. 3A — total protein and B — extracellular protein). Both the extracellular and total protein extracts stored in buffer 1 displayed a statistically significant % decrease in protein concentration after 1 week of storage at − 20 °C (P < 0.003 for both extracts). Total protein extract stored in buffer 2 showed a significant % decrease in protein after 1 month (P < 0.002) and extracellular protein stored in buffer 2 showed a significant % change in protein concentration after 3 months (P < 0.004).

The stability of faecal proteases in total protein extraction was also determined. All samples demonstrated a significant correlation between length of storage and the % change in protease activity (Fig. 3C — total protein and D — extracellular protein) suggesting that protease activity in all samples was affected deleteriously by the length of storage. The protease activity in the total protein stored in buffer 1 and buffer 2 displayed a significant decrease after 1 week of storage (P = 0.003 and P = 0.003 respectively) whereas the extracellular protein protease activity displayed more stable protease activity, which only began to show significant changes after 1 month of storage in buffer 1 (P = 0.004) and after 3 months of storage when stored in buffer 2 (P = 0.004). These data indicate that both buffers are only appropriate for storing total protein isolated from faecal samples for a short period of time (1 week) before they begin to show changes from their original activity and bias further function-based analyses. Extracellular proteases, those not extracted with bead-beating, appear to be more stable in these buffers.

### 3.4 The effect of freezing temperature on whole native faecal samples and reproducibility of protein concentration and protease activity

**Fig. 4:**
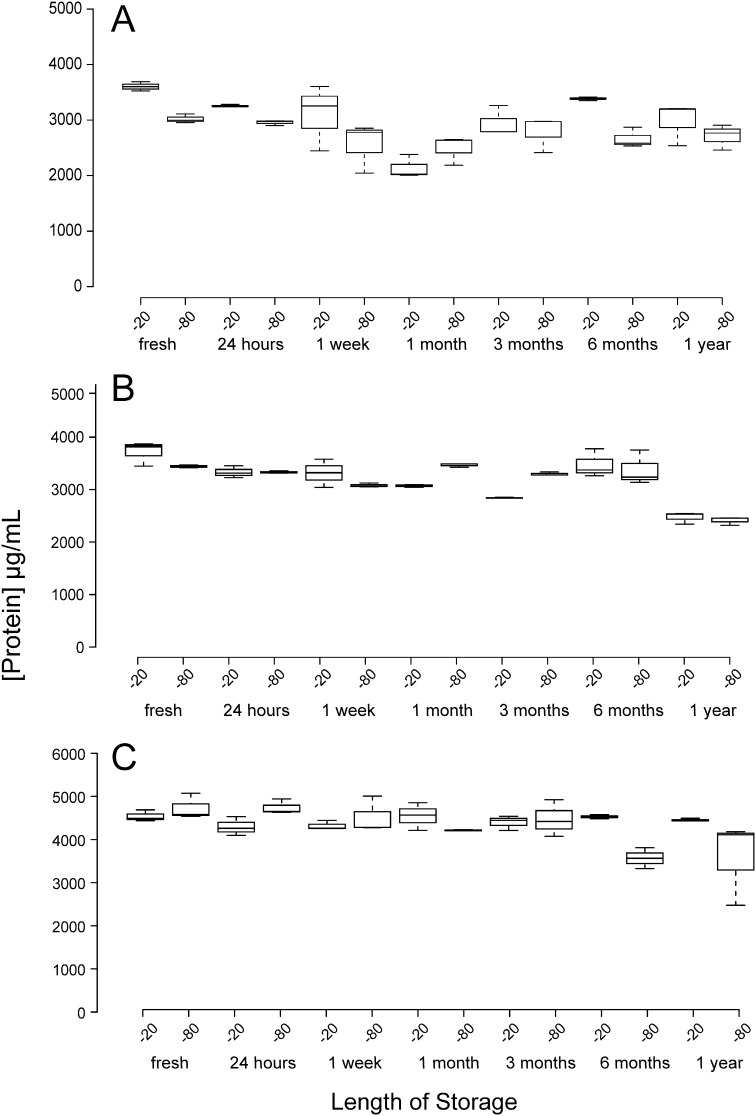
Effect of freezing temperature of whole faecal sample protein concentration estimates. The effect of freezing temperature and the length of storage on whole faecal samples and their protein concentration from three individuals; A, B and C. Boxplots indicate the results of triplicate data, centre lines show the medians; box limits indicate the 25th and 75th percentiles; whiskers extend 1.5 times the interquartile range from the 25th and 75th percentiles. The data were analysed by Wilcoxon signed ranked tests to assess whether protein concentration deviated from the concentration measured upon fresh extraction.

**Fig. 5:**
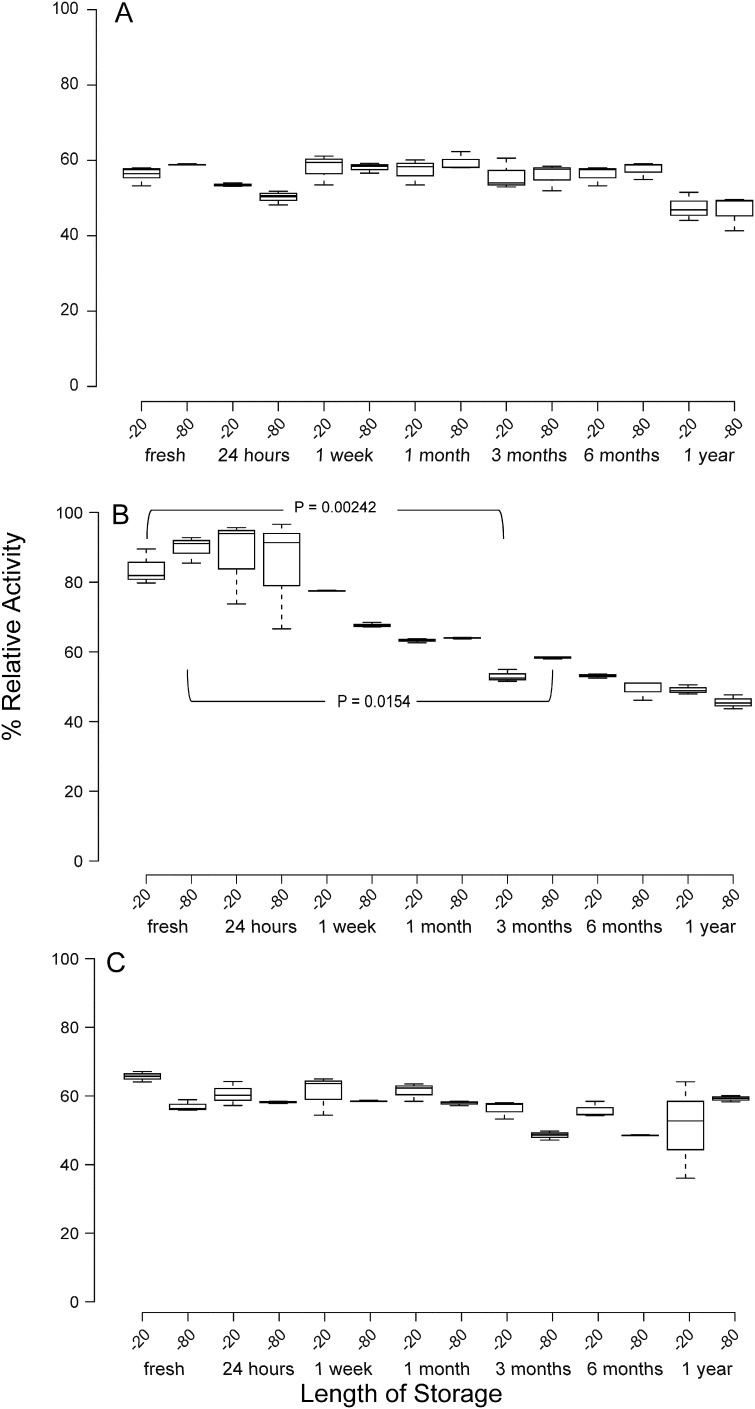
Relative protease activity (%) after protein extraction of faecal samples stored for one year at − 20 °C and − 80 °C. The effect of both freezing temperature and the duration of storage of faecal samples from three individuals; A, B and C on protease activity after normalisation to 1 mg/ml protein following protein estimations. Boxplots indicate the results of triplicate data, centre lines show the medians; box limits indicate the 25th and 75th percentiles; whiskers extend 1.5 times the interquartile range from the 25th and 75th percentiles. % relative activity was determined using 1 mg/ml proteinase K incubated with the same substrate for 2 h. The data were analysed by Wilcoxon signed ranked tests to assess whether protease activity deviated from the activity measured upon fresh extraction. The time at which activates became significantly different to original activity (fresh samples) are indicated along with their respective P-values.

Protein concentration was not affected by length of storage for any of the samples collected from the three different individuals (Fig. 4) nor was it affected by storage temperature suggesting that faecal protein remains stable when frozen as part of the original biological sample at sub-zero temperatures. Two thirds of samples displayed no significant alterations in protease activity following normalisation to 1 mg/ml protein over the entire period of the study at both of the storage temperatures (Fig. 5A and C), however, samples collected from individual 2 exhibited a significant change in proteolytic activity following three months of storage at both sub-zero temperatures (Fig. 5B).

## 4 Discussion

The gut microbiota has an enormous diversity in terms of both phylogeny and functionality (Eckburg et al., 2005, Lozupone et al., 2012), therefore a diverse array of techniques including ‘omic’ approaches as well as function-based assays need to be implemented if we are to fully understand the impact they are having on us, as their hosts. However, as highlighted by Flores and colleagues, in their study of the reproducibility of β-glucuronidase and β-glucosidase enzymes in faecal samples after extraction and storage (Flores et al., 2012a, Flores et al., 2012b), researchers cannot begin a study without first assessing the parameters that may be affecting the gut microbiota and the molecules they wish to measure.

The aim of this work was to assess the stability of protein extracts isolated from faecal samples over time, to deduce which buffer composition was the most appropriate for long term storage, and whether freezing faecal specimens before processing, was appropriate for maintaining protein and protease stability. Due to the overall aim of measuring the protein content there could be no extraneous input of proteins. Therefore, the composition of the buffer could not include carrier proteins such as BSA which normally ameliorates the problem of low-level binding to storage vessels, metal chelators such as EDTA as this could affect the activity of some proteases, and protease inhibitors because they will inhibit the activity of the proteases which we wish to study (Ji, 1973). It was also found that the reducing agent dithiothreitol (DTT), which prevents oxidation of cysteines, severely skewed the protein concentration measurements so it could not be used for further experimentation (Ji, 1973). Consequently, it was decided that the final buffer compositions to compare would be simply PBS supplemented with 0.05% (w/v) NaN_3_ (buffer 1 compared to a 20% v/v glycerol–PBS buffer also supplemented with 0.05% (w/v) NaN_3_ (buffer 2). NaN_3_ is a potent antimicrobial and so it's inclusion in the buffer was essential for activity as a broad spectrum anti-microbial to prevent microbial contamination of faecal protein extract. It was shown that NaN_3_ did not interfere with protease activity of the samples (Fig. 2) and was successful in preventing microbial contamination evidenced by plating faecal protein extracts onto nutrient rich media and no bacterial growth occurring (data not shown). Therefore it was concluded that NaN_3_ was a useful component of a buffer for storage and maintenance of protease enzymes extracts in a complex biological sample.

Beadbeating is now a common feature used to lyse bacteria and increase the degree of lysis, especially when isolating DNA from complex systems such as soil or faecal material. The number of bead beating steps for optimal protein extraction was deduced by bead beating a sample from 1 to 6 times. The protein concentration and protease activity were measured for each of the bead beating samples and it was deduced that 3 bead beating steps was adequate to get maximum protein out of a sample without the addition of lysis enabling buffers (Fig. 1).

It is common to try and stabilise extracted proteins in some form of buffer in order to maintain activity, e.g. commercial proteinase K is supplied in a buffer containing 40% (v/v) glycerol 10 mM Tris–HCl, pH 7.5 and 1 mM calcium acetate. Thus we tested similar buffer systems, but excluded any stabilising proteins such as BSA, which would interfere with protein quantification. Buffer 1 and buffer 2 were compared over a period of 1 year with protein concentration and protease activity recorded immediately upon extraction, after 24 h stored at − 20 °C and then after 1 week, 1 month, 3 months 6 months and finally 1 year to determine their expediency as buffers for protease storage. All samples in both buffers exhibited a negative correlation between both protein concentration and protease activity over time (Fig. 2) and no sample displayed repeatable protein levels or protease activity after 1 year nor 6 months of storage suggesting it would not be advisable to use either buffer for long term storage of protein extract for protease based studies. Assessment of shorter term storage revealed that after 1 week of storage total protein extract began to significantly decrease in concentration in both buffers, but extracellular protein remained more stable for 1 month in buffer 1 and 3 months in buffer 2 suggesting that buffer 2 was more suitable for longer term storage of extracellular protein, but also that extracellular proteins are more stable than intracellular proteins perhaps because proteins that are secreted from cells are adapted to the harsher, less controlled environment of the gut lumen.

The observed decrease in protein concentration is likely due to changes in temperature, low-level binding to the storage tube and probably proteolytic degradation by proteases within the sample. When the protein samples increase in temperature, upon thawing, it is likely that the proteases present will also be contributing to the decrease in protein concentration by destroying proteins. Protease activity, however, was also diminished in all samples over the period of a year, in fact, protease activity from the total protein extracted began to deteriorate after just 1 week of storage. The most likely cause of this drop in activity is the loss of their native structure due to denaturation due to sub-zero temperatures affecting protein structure (1990) and perhaps proteolytic degradation of other proteases. Therefore, despite the presence of a cryoprotectant, the enzymes can still be rendered inactive. The extracellular protein maintained activity for up to 1 month when stored in buffer 1 and for 3 months when stored in buffer 2. Thus the glycerol is clearly playing a part in maintaining extracellular protease activity, but this also indicates that the extracellular protein is more robust than intracellular protein. It is likely that an increased concentration of glycerol would be more effective in maintaining both protein and consequently protease activity of faecal samples, however, this would interfere with other assays (as well as the BCA assay and other protein quantification assays) and so is not ideal for studies aiming to evaluate protease activity.

A lot of studies involving the human gut microbiota involve the storage of whole faecal samples and different storage conditions can sometimes affect experimental outcomes. The effect of storage condition on faecal samples and concentration of other microbial molecules as a result of freezing have been studied (Flores et al., 2012a, Flores et al., 2012b, Khan et al., 2002), but not the effect of freezing entire faecal samples and its impact on total protein or relative protease activity. We found that for all samples isolated from the 3 individuals there was no significant reduction in protein concentration even after a year of storage at both − 20 °C and − 80 °C (Fig. 3). Protease activity remained stable for the entire study period as well, even when faecal samples were stored at either temperature (Fig. 5) for two of the individuals studied. One sample however, displayed a reduction in activity after 3 months at both storage temperatures (Fig. 4B) although it is noteworthy that this sample displayed higher protease activity than the other two samples suggesting that self-proteolysis was playing a part in this reduction of protease activity.

Therefore, from this study it could be advisable that for future studies of protease activity in the human distal gut, if protein is extracted, a buffer comprising of cryoprotectant along with an antimicrobial, both of which do not alter protein concentration or protease activity, and storage at − 20 °C are suitable. If samples are required for extensive analysis which is likely to take more than 1 day, samples can be extracted in a glycerol–PBS buffer in a neutral pH and will remain stable for up to 1 week.

However, protein concentrations and protease activity measurements are more reliable when carried out in the analysis of frozen faecal samples. Following analysis of the results of this study it would be recommended that upon retrieval of faecal samples, 1 g samples should be dispensed into appropriate, sterile storage vessels for future protein extractions. This approach will be more appropriate for storage in terms of space and importantly will help avoid repeat freeze thaw cycles of the entire sample and decrease the chance of contamination. Also, care must be taken with samples exhibiting significantly higher levels of protease activity compared to the rest of the cohort after storage for longer than 3 months as samples kept this long may not be representative of the gut microbiota from that individual. Though to conclude, human faecal samples offer a highly reproducible means of accessing protease activity of the human distal gut even after long periods of storage at sub-zero temperatures.

The following are the supplementary data related to this article.

Fig. S1 Schematic diagram highlighting the methodology for isolating extracellular and total protein for analysing the most appropriate buffer for storage and activity measurements of proteases isolated from 3 individuals and stored for one year, with analysis at various intervals.

Fig. S2 Schematic diagram highlighting the methodology for assessing the effect of one year of storage of faecal samples for protein and protease activity measurements and various intervals throughout the year.

## Competing interest

The authors declare that they have no competing interests.

## Author's contribution

JRM and LSM, conceived and designed the study. LSM performed the experimental work and wrote the first draft of the manuscript. JRM and LSM edited and completed the final draft. All authors read and approved the final manuscript.

## Figures and Tables

**Table 1:** Buffer composition.

Buffer	Buffer composition
1	PBS, 0.05% w/v NaN_3_
2	PBS, 10% v/v glycerol, 0.05% w/v NaN_3_
