# Supplementary figures and images for: Assessing the impact of long term frozen storage of faecal samples on protein concentration and protease activity
Source: J Microbiol Methods. 2016 Apr;123:31–8. doi: 10.1016/j.mimet.2016.02.001 (PMC4819717; doi:10.1016/j.mimet.2016.02.001)

Figure S1

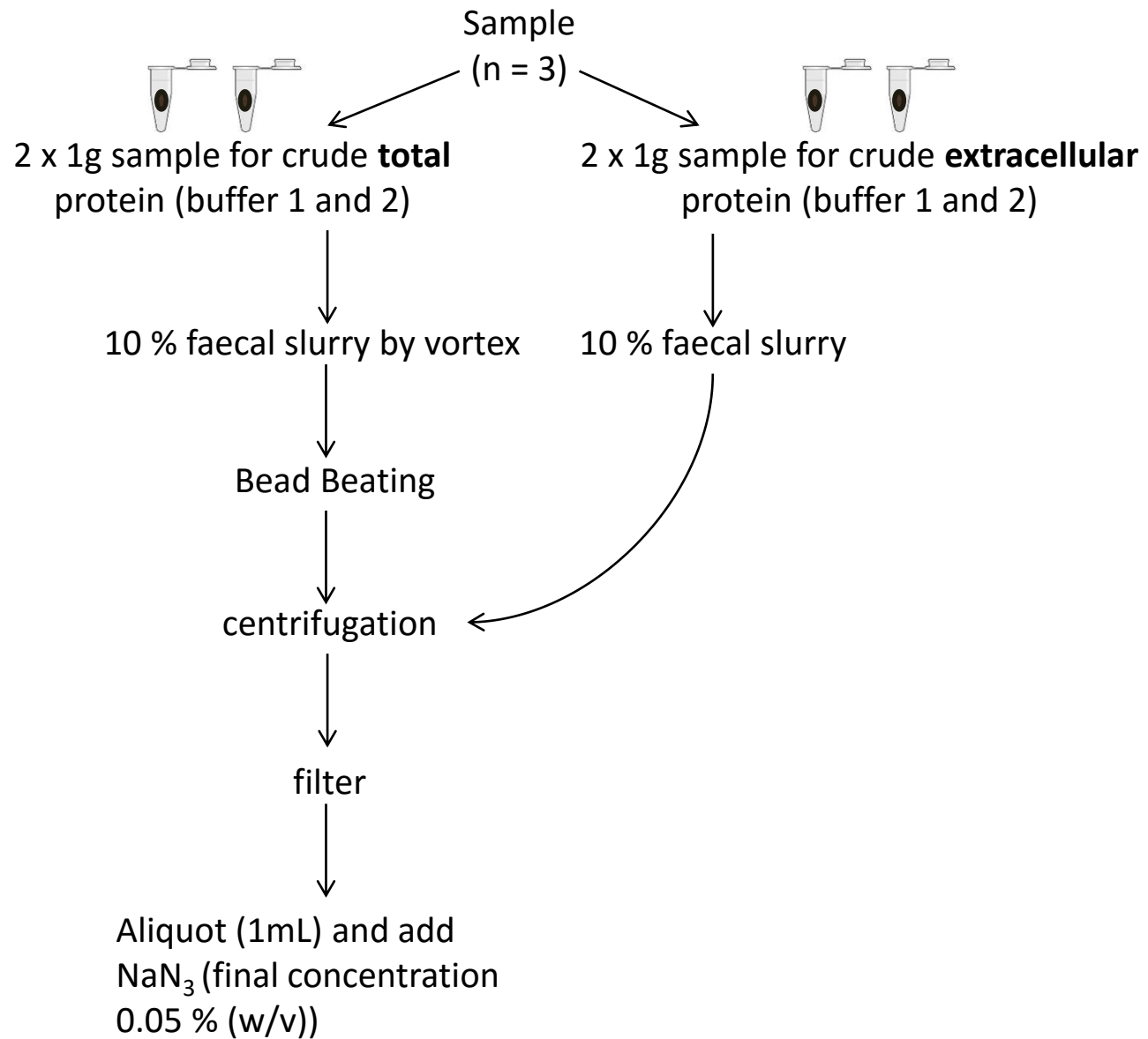

Supplement: Fig. S1 — Schematic diagram highlighting the methodology for isolating extracellular and total protein for analysing the most appropriate buffer for storage and activity measurements of proteases isolated from 3 individuals and stored for one year, with analysis at various intervals. [file mmc1.pdf]

Figure S2

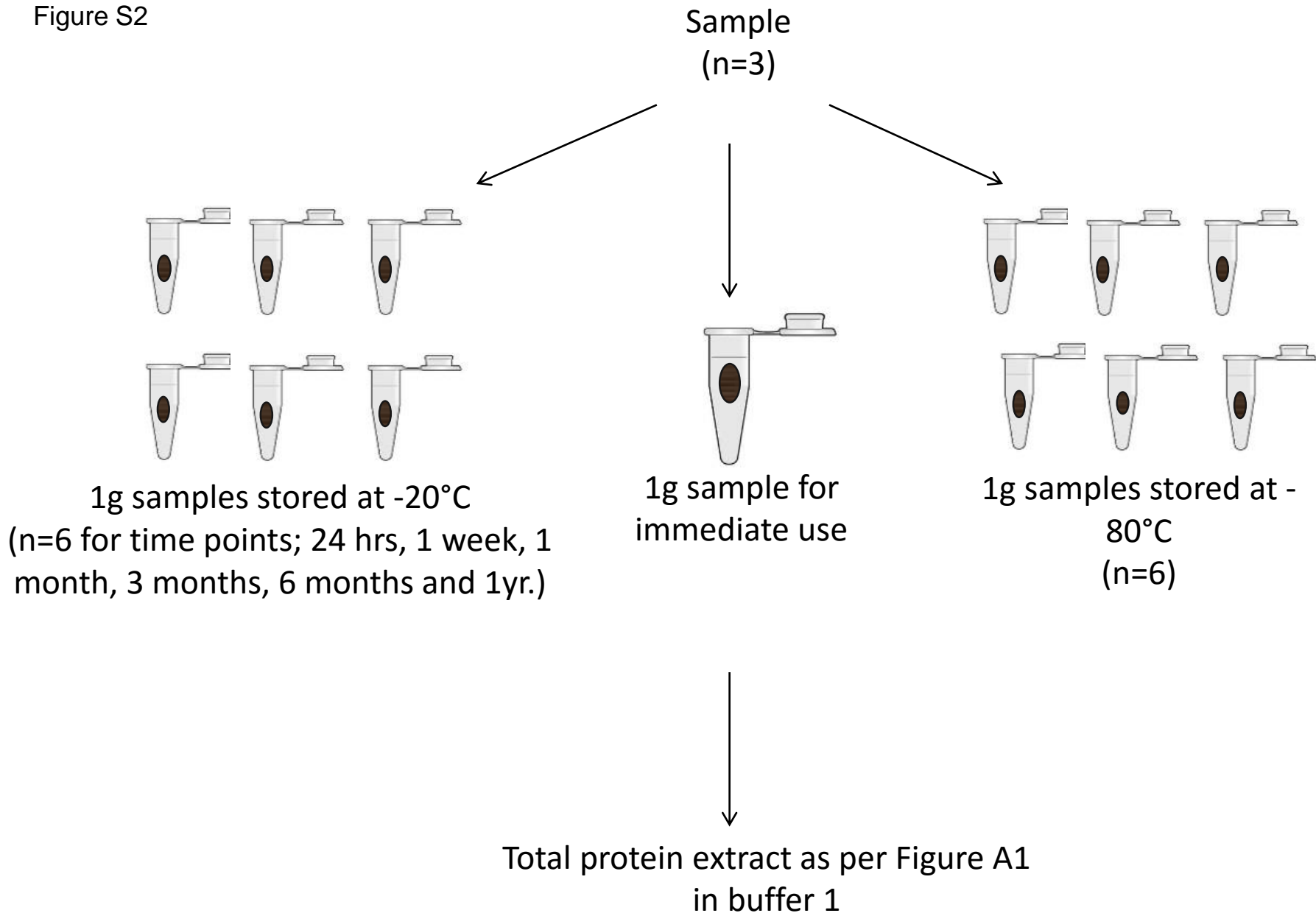

Supplement: Fig. S2 — Schematic diagram highlighting the methodology for assessing the effect of one year of storage of faecal samples for protein and protease activity measurements and various intervals throughout the year. [file mmc2.pdf]
